# Dynamics of Natural and Homœopathic Science

**Published:** 1896-06

**Authors:** B. Fincke

**Affiliations:** Brooklyn, N. Y.; Bellasylva, Pa.


### DYNAMICS OF NATURAL AND HOMŒOPATHIC SCIENCE.

(Thoughts on the essay of Dr. C. Kunkel, in Kiel: Are Matter and Force Cause and Effect ? *)

B. Fincke. M. D., Brooklyn, N. Y.

Goethe's dictum on the natural research of his time, "that indeed nothing could be said against its exactness in detail, but that the fundamentals are passed over too lightly," which the author places at the head of his essay, is inestimable. It is a pity that the great man paid no attention to another great man growing up side by side with him, viz., Hahnemann. But we know that the friend of Goethe, the celebrated Doebereiner, rejected Hahnemann's discovery of the active principle of the alkali, called by him Causticum, which has become one of the most valuable remedies in the homœopathic materia medica. So likewise the rare and costly Platinum, which came to Doebereiner's hands through his friend Goethe, and led him to the discovery of Platina-black and the invention of the Platina igniting machine, grew in the hands of our own Stapf to one of the best homœopathic remedies in acute and chronic diseases, unknown to the chemist from whom it came. The reason of this negligence of those great men of science lay in their acknowledgment of Materialism under the disguise of the then prevailing Rationalism, which also Hahnemann followed till he found another view answering his research in medicine, the dynamic philosophy founded upon the life-force animating all matter. Goethe's "Farbenlehere" confirms the idea of his Materialism, and his refutation of Newton roots in it because Newton's fundamental ideas were decidedly dynamic, and conform with Hahnemann's dynamicism, to which this philosopher arrived at the hands of facts which grew out of the potentiation

* Sind Stoff und Kraft, Ursache und Wirkung. Ein Beitrag zur Arzneifrage. 2 Auft. Kiel und Leipzig: Lipsius & Fischer. 1894.

of substances for the purpose of assimilating them to the life-force which animates everything, therefore also the human body. Thus it appears that the dictum of Goethe at the head of our essay goes against himself, for his aggression of Newton is so bitter because he himself went over the fundamentals of all natural science, the universal gravitation, the origin of which Newton left to every one studying it, without fastening it to a materialism such as dominates the researches of the scientists up to the present day.

The "Unity of Life" is curiously maintained by the natural philosophers, physicians, physicists, and chemists of the day, but for reasons which on account of the incongruity in thinking cannot bear close scrutiny. For they try to explain life and especially organic life by physical and chemical laws which, however, according to their views only find their application upon what they call dead matter, because those laws are deduced from the behavior of matter to each other, naturally or experimentally. This contradiction has not come to their consciousness, since they do not acknowledge any other forces than physical and chemical forces. They close their eyes to other forces or merely put them aside in order to take them up again when the science will be so far advanced in the future ages, to remember the material stowed away for eventual future use. Nothing indeed can be said against this. These scientists, however, should be careful not to distrust, slander, and hinder efforts which aim at no less than to assign now to these forces their proper places as well as those of physics and chemics themselves in general science.

Naturally the "Unity of Life" comprises simultaneously the so-called material and spiritual world. The life is the motion which penetrates everything and by means of matter mediates the phenomena which are ascribed to the realization of their underlying laws. The physical and chemical forces of inorganic things are of the same nature as we observe in the organic world, but they are more simple, and therefore easier to deduce and demonstrate, than those of the complex organic bodies. The unity of life can certainly not be subject to any doubt. Life is a series of motions from the lowest to the highest, from the simplest to the most complex with eradiation of infinitely many side-series which in all possible angles deviate from the line of the main series. But motion, what is motion? Change of place. Change of place of what? Change of something which moves of itself or is moved. But no matter moves of itself. How uncertain is this concept of motion! From the appearance and from the experience it is accepted without much ado that it must always be a body which is subjected to the motion. But does not also the spirit move? Are not the thoughts in constant motion, in waking and dreaming? By the motion all things are connected with each other. By the motion they act upon each other. Without motion nothing can be conceived as existing. The motion, therefore, is more than a mere property of something else, it is something itself and certainly something which is most difficult to understand, because it enters into the composition and existence of all things without being a material thing itself, and without it these things are not at all thinkable. Unfortunately there has been attached to the word "Motion" always a low concept in the science of mechanics, which has to deal only with dead inanimate objects and their action upon one another. But always at the same time the universal motion which pervades the universe in all its large and small parts, called "Universal Gravitation," the universal attraction which is the realization of the fundamental principle of "Universal Assimilation" or "Homœosis," lying at the bottom of it, is forgotten or neglected or unknown.

If now by the Hahnemannian potentiation we find that in all substances exists a force which of different quality only after a peculiar manipulation under elimination of all material appears and is assignable only by its application to the human (animal) organism, we immediately arrive at the fact that only by an inert vehicle this force can be evolved from the substance. This points to the fact that the substances, aside of their physical and chemical forces, contain still other forces unassignable by these departments of science which can be called medicinal forces, since they can be recognized only by the pathogenesis and pathopoësis of the organisms, but never by mere physical and chemical experimentation. The present conception of natural science, therefore, has no right to fasten the life of organic bodies upon the procrustes bed of physical and chemical laws concerning matter as mass. This matter serves only to furnish to the organism the material for its growth and development. Though this material naturally is subject to these laws also, it does not follow that what is characteristic of the inorganic stone and earth in physics and chemics is necessarily also the characteristics of the organic body in dynamics. In relation to this, which, and though it comprises in itself the physical and chemical laws, we may ascribe life also to matter, since in its potentiation it must contribute to the organization of the higher organic bodies. Therefore, instead of degrading the organism by the appearance and the necessity of the realization of the physical and chemical forces in it to a lower value, he forces of the so-called dead and inanimate bodies have to be raised to a higher grade by the concept of life. And thus we arrive at the idea that what is called motion comprises the concept of life in itself as the continually-acting force in the universe which refines the matter by potentiation and utilizes it for the formation of organisms.

In this regard no objection can be raised to the conception of a unity of life. On the contrary, it is a great progress in the modern, natural science, which, without knowing or willing it, follows imperceptibly in the wake of the Hahnemannian dynamic philosophy.

This is just the error into which they fall, that they consider the organism as though a very complicated mechanism or machine which can be taken asunder and in all its parts treated *ad libitum*. For the machine is only in so far valuable as it fulfills the purpose for which it was made. The decomposition of it disturbs its unity of action and with it its purpose. The parts of the machine have every one its own life, according to the quality manifesting itself in its action. Just so the organism. Without the unity of life it is no more an organism. It is indeed a complicated mechanism, but a great deal more. For in it are deposited the higher organs, the life-force, the soul and the spirit which manifest themselves in their activity by means of the mechanism of the organism. Nothing can illustrate this better than the Hahnemannian concept of the life-force. Only it should not be conceived as an abstract something which independently like a king sits upon his throne and rules his subjects. The life-force is the blossom of the mechanism, and cannot be missed in the living organism on account of the mentioned necessary unity of life, the force of life of the whole organism in its entire unity. May the adherents of the physico-chemical school cease to acknowledge the life-force and throw it away into nothingness, as they imagine: *naturam expellas furca, tamen usque recurrit.* The poor, slandered Genoveva thrust into the wilderness, who must subsist upon the products of Homœopathy, will be vindicated, and like this be reinstalled in her full rights.

Therefore, indeed, the conception of the unity of life excludes the expression of a dead nature, for everything moves or is moved—*i. e.*, everything lives.

The declaration of the action of the mineral waters which owe their virtue to something else than the matter contained in them in inappreciable quantity, in the essay before us is quite appropriate to lead the natural philosophers the right way, since it is clear and admitted by them the amount of matter they hold cannot be the characteristically healing power in them, the peculiar animating something which these waters undoubtedly possess. Here our fluxion-potencies present an analogy and offer a practical solution of the problem. After the medicinal substance has been subjected to a thorough succussion in the initial potencies prepared according to Hahnemannian rule, it is submitted for a long time to a slow continuous stream in the proportion of one to hundred, a process which decidedly excludes every idea of attenuation of matter. At least after the twelfth centesimal potency every scientific test fails to reveal the presence of any matter.

The proof of the efficacy of these fluxion-potencies lies in their application upon the healthy and sick organism. The

*modus operandi* of this process is not the attenuation of matter, but the transference of the medicinal forces contained in the crude medicinal substances upon an inert vehicle. The process potentiates the inert vehicle and renders it medicinal in the direction of the original substance used. As any mechanical force is transmitted by machinery for the work intended, so the undulation of the fluxion carries the medicinal force to all the infinitesimal parts of the fluid vehicle employed and makes out of them potencies powerful to sicken or to heal as the case may be.

Now the mineral waters in question are fluxion-potencies prepared by Nature by means of a continuous fluxion for many miles. The medicinal forces of the matter over which this takes place yield their power by transference to the undulations of the flowing water; and afterward at certain places they show the healing qualities, which are made use of by drinking and bathing. How many medicinal forces enter the current it is impossible to say; but some of them must be predominant, because peculiar characteristic healing qualities, and also the reverse, are observed from the different waters. That the wave-motion of the waters supplants the Hahnemannian succussion and facilitates the potentiation is probable. In our fluxion-potencies the succussion has been made use of only in the initial potencies. In the higher potencies the simple undulation of the slowly-flowing water has been sufficient to transfer the medicinal force throughout the whole mass of vehicle.

The learned hypotheses which have so far been advanced for the explanation of the action of the mineral waters in question, which escapes the acumen of physical and chemical investigation, may do all honor to their proponents, but they fail to give a practical result which leaves no reason for speculation and satisfies the most rigorous philosophical method of scientific research. But, of course, those adherents of the physico-chemical school must study Hahnemannian Homœopathics if they want to approach a solution of the problem before them. Our potentiation by fluxion, however, furnishes a simple type how forces can be transferred through masses of fluid vehicles with ease and certainty and preserve the character of the medicinal force of the original medicine substance intact to the highest degrees. The proof can only be furnished by the homœopathic argument, which consists in the administration of the potencies upon the human organism in proving and healing. This proof has been given, for these fluxion-potencies are now in constant use in acute and chronic diseases by the homœopathicians for the last thirty years. But our opponents, the scientists *par excellence*, are so ignorant in regard to homœopathic science that it is no wonder that they cannot find the explanation of the efficacy of the mentioned mineral waters. They turn around and around to escape the true solution of the problem, which can only be reached by the homœopathic potentiation.

The mineral waters in question, then, are fluxion-potencies obtained by Nature through the instrumentality of flowing water over medicinal substances. Through flowing along large distances for miles and miles, the substances coming in contact with the water are potentiated—that is, the medicinal forces contained in them are taken up by the water and transferred through the undulation to immense masses of water serving as vehicle, so that when they appear at the surface of the ground as springs, they exert their healing or sickening action as the case may be, which are recognized by their administration to the human body. Our potencies pass also along a way of many miles in the slow exit of the vehicle from the potentiating vial. The undulation of the flowing water which by its gravity seeks the lower level is the necessary element for the transference of the medicinal forces while spread through and penetrate all the infinitesimals of the moving vehicle. Our potentiation differs from the natural one as it limits itself to the potentiation of one single substance, whilst, in the natural one of the mineral waters (so-called), a multitude of substances is concerned. Thus the value of our potentiation depends upon its simplicity as a scientific experiment. There is nothing than the substance acted on, and the undulating vehicle moving according to the laws of gravitation. The inductive experiment of applying the thus obtained potencies in health and disease, leads us to the undoubted fact in nature proved by physics and chemics, that it is not the matter which is transferred by a supposed but erroneous infinite divisibility of matter through the inert vehicle, but the forces dwelling in the matter and the proposition, therefore, that all matter contains medicinal forces, aside from physical and chemical ones, is not a hypothesis, but an established fact to be accepted as fundamental in general science.

That the powerful succussion enjoined by Hahnemann, who thought in it to find the explanation of development of medicinal force has that significance and the great value assigned to it is very doubtful, because efficacious remedies are obtained by gentle motion of the fluid vehicle by simple fluxion.

But it is entirely beyond doubt that—*e. g.*, a Cm Lachesis, which exerts its potency upon the healthy or sick human body, cannot be derived from anything but the original drop of the poison obtained from the living snake. This small drop has the immense series of hundred thousand potencies behind itself, with all the series which may be derived from every drop of the vehicle-series *ad infinitum*, and every one is potent to act upon the human body according to homœopathic law.

Inversely, all those infinite series of potencies which are medicinal forces can be thought to be contained in the original drop used for potentiation, and there is no other way to think of it, for during the potentiation nothing was concerned in it than the drop originally used and the inert vehicles. Nay, the forces obtained from the drop are by no means exhausted in it, and probably still more infinite series have been in it which could not find development.

According to the most approved scientific methods the last molecule of matter had disappeared already in the twelfth centesimal potency, nay, somewhat before it, though this limit has been appointed to it only by mathematical calculation of the last molecules of another substance, and this has been adduced as a proof that all the potencies claimed after this arbitrary limit of potentiation are mere figments in the brains of those thinking differently. An experience of more than fifty years by hundreds of homœopathicians, obtained by the administration of high potencies upon the sick, and by proving them upon the healthy, is as nothing to these stubborn minds, who find virtue in matter only, and calumniate force as being subservient to matter. It is useless to try to convince these sceptics, who cling to their scepticism as the wisdom of the age, and call science what is only short-sightedness of unscientific methods. If experience and experiment and judicious observation of facts form the fundament of science, the fact of the efficaciousness of high potencies must be acknowledged as far as these necessary conditions of research reach. Thus far the terminus of efficaciousness of high potencies has not been reached. Whether there is one must be left to future investigation, and if the infinite divisibility of matter must be denied, one of force might indeed be assumed till that terminus is found. But what is known for certain is enough to maintain that here we have to do with peculiar forces, which thus far have been denied by the chemists and physicists, and also by the physicians, forces which are dwelling in matter of various kinds; medicinal forces which by means of proper manipulation can be transferred upon the inert vehicles used for their development, and furnish powerful instruments for healing the sick according to homœopathic principles. The potency depends not alone upon the dequantitation of matter, but also upon the quantitation of the medicinal forces residing in it, upon their distribution in great masses of vehicle which assume the quality of the matter from which it has been derived, and the relation of it to the organism in health and disease, and its sensitivity.

The doctrine resulting from these observations, experiences, and experiments is so strange to the mind used to consider only matter as real that it rejects it either with ridicule or indignation. Both these critical objections, however, are no valid arguments against it, and are not worth the least attention. Its immense value appears when this doctrine, which we owe to Hahnemann, is applied to all conditions of matter. If in our potentiation the inert vehicle is necessary to bring out the medicinal forces of matter, and medicate it for use upon the living organism, the thought presents itself very readily: is not all matter the recipient and keeper of force, and enables it to pass from one part of matter to another as the gravitating force which is transferred through all matter of the universe, and working the wonders which science is constantly revealing in its incessant striving for greater knowledge and wisdom? Are not the physical and chemical forces of matter just similar forces which by transference from one part to another produce the manifold phenomena forming the objects of the different departments of science which it is trying to recognize and utilize? We have already the science of dynamics in physics in contradistinction to statics which is nothing else but disguised dynamics, for nothing is in an absolute state of rest. The transference of force from and through matter is illustrated sufficiently by mechanics. The machinery serves as a vehicle to direct the forces developed from matter upon the masses to be moved.

Force is not conceivable without motion. Motion is the necessary condition of force. Light, electricity, magnetism are forces produced by the hands of Nature. They are potentiations on a grand scale, of which our potentiations are only a simple type. Even they, however, can be further potentiated on the fluxion method, and made applicable to healing in the form of high potencies. According to modern science, they are modes of motion, but it is not said what that motion is. It is motion of force, and moving force is life, and life resides in everything existing, and this is the unity of life in the universe spoken of before. Truly, the Hahnemannian potentiation is the grandest idea which the nineteenth century, the end of which we are now permitted to see, has brought forth. All the great and numerous inventions and discoveries dwindle to nothing against the conception of a universe vouchsafed by it, which is as great as simple, and agrees with the reasoning of common sense and a sound understanding and a scientific wisdom. The life-force is given from eternity and lasts through eternity. It is carried through the phenomena of matter from one body to another, large and small, throughout the universe, rooting in the vast principle of gravitation. This is the lifeforce which dwells in all and everything in various innumerable degrees and directions, and is brought to the highest perfection in the wonderful organism of man.

We, however, here have to do only with the medicinal forces which we use to heal the sick according to the eternal principles of pure homœopathics as revealed and taught by Hahnemann. But of this the ruling powers of natural science and medicine, and also the majority of our own profession, do not want to know anything. They turn with disdain from the acceptance of the Hahnemannian ideas which they do not take pains to understand, because they militate against the accepted tenets of materialistic philosophy. They will not acknowledge the great fact of the almighty source of life-force, and please to be considered as gods themselves whilst they live. They are actually adored as saints! Hence they try to force any science opposed to their way of thinking out of existence, because it is so unpleasant to be reminded again and again that their materialistic philosophy resembles the terrible image of Nebucadnezar standing on feet of day. Even the good Newton took care not to derive gravitation from matter, and left it to every one to discern its cause. But it is all in vain to convince those who do not want to see. How many human beings must perish before their time of allotted life, before the great lights of science will take proper notice and accept the beneficent doctrine of Hahnemann and execute it, instead of running after the false lights of botched methods, which, supported by the political forces, are forced upon poor humanity as the *ne plus ultra* of medical wisdom. If they were reasonable, the little pamphlet of Dr. Kunkel would give them pause. But it is all in vain. Only the successful cures, according to the Hahnemannian art, multiplying as the art and science of homœopathics progresses in its further cultivation on our own ground, can promise a better future for suffering man.

Certainly the law of mechanical acquivalence is decisive for the organic life, though it comprises the law of quantitation; but it is so just as well for inorganic life on account of the unity of life claimed by general science now, as mentioned above.

This law of mechanical acquivalence is nothing else than the law of proportionality applied to medicine in Hahnemann's similia similibus curantur, and in the relation to the sensitivity of the organism.

Under no conditions, therefore, can matter be looked at as the cause of force, for this is the grossest materialism, leading back into the wilderness of savage life. No! The Hahnemannian idea of potentiation applied to the acknowledged unity of life is the correct view, viz.: That matter is only the vehicle of force, hence inert *per se*. Wherever the force comes from may be revealed to those who after death care for it, if they are not content to know already during their life on this earth, that it comes from God, the creator and preserver of the universe.

But whence does matter come? No doubt from the same Almighty source; but what, according to the idea of potentiation matter itself is, is a nut which we must leave to the materialistic philosophers to crack, as it is their undoubted duty if they make it the supreme power governing all science.

*Ceterum censeo macrodosiam esse delendum.*

B. Fincke.

Bellasylva, Pa., Sept. 4th, 1895.

